# Labelled drug-related public expenditure in relation to gross domestic product (gdp) in Europe: A luxury good?

**DOI:** 10.1186/1747-597X-5-9

**Published:** 2010-05-17

**Authors:** Luis Prieto

**Affiliations:** 1 Interventions, Law and Policies Unit, European Monitoring Centre for Drug and Drug Addictions (EMCDDA), Cais do Sodré, 1249-289 Lisbon, Portugal; 2 London School of Hygiene & Tropical Medicine, Keppel Street, London WC1E 7HT, UK

## Abstract

"Labelled drug-related public expenditure" is the direct expenditure explicitly labelled as related to illicit drugs by the general government of the state. As part of the reporting exercise corresponding to 2005, the European Monitoring Centre for Drugs and Drug Addiction's network of national focal points set up in the 27 European Union (EU) Member States, Norway, and the candidates countries to the EU, were requested to identify labelled drug-related public expenditure, at the country level. This was reported by 10 countries categorised according to the functions of government, amounting to a total of EUR 2.17 billion. Overall, the highest proportion of this total came within the government functions of Health (66%), and Public Order and Safety (POS) (20%). By country, the average share of GDP was 0.023% for Health, and 0.013% for POS. However, these shares varied considerably across countries, ranging from 0.00033% in Slovakia, up to 0.053% of GDP in Ireland in the case of Health, and from 0.003% in Portugal, to 0.02% in the UK, in the case of POS; almost a 161-fold difference between the highest and the lowest countries for Health, and a 6-fold difference for POS. Why do Ireland and the UK spend so much in Health and POS, or Slovakia and Portugal so little, in GDP terms? To respond to this question and to make a comprehensive assessment of drug-related public expenditure across countries, this study compared Health and POS spending and GDP in the 10 reporting countries. Results found suggest GDP to be a major determinant of the Health and POS drug-related public expenditures of a country. Labelled drug-related public expenditure showed a positive association with the GDP across the countries considered: r = 0.81 in the case of Health, and r = 0.91 for POS. The percentage change in Health and POS expenditures due to a one percent increase in GDP (the income elasticity of demand) was estimated to be 1.78% and 1.23% respectively. Being highly income elastic, Health and POS expenditures can be considered luxury goods; as a nation becomes wealthier it openly spends proportionately more on drug-related health and public order and safety interventions.

## Findings

Producing estimates of drug-related public expenditure was one of the many targets set by the former (2005-08) European Union's (EU) drugs action plan [1]. In this light, the European Monitoring Centre for Drugs and Drug Addiction (EMCDDA) [2] launched in July 2008 a publication focusing on Drug-related public expenditure in Europe [2]. Testing a new methodology of combining 'labelled' and 'unlabelled' expenditure, the report presented estimates of how much European governments spent in 2005 on the drugs problem.

'Labelled drug-related expenditures' are defined as the direct ex-ante planned spending that reflects the voluntary engagement of the state in the field of illicit drugs. Direct public expenditures explicitly labelled as 'drug-related' can be easily traced back by exhaustively reviewing official accountancy documents such as national budgets and year-end reports. The national budget is a comprehensive document, encompassing all government revenue and expenditure, so that the necessary trade-offs between different policy options can be assessed. The government's draft budget is submitted to the national parliament for review and approval prior to the start of the fiscal year. The final approved budget, and related documents, include a detailed description of each revenue and expenditure programme (i.e. the particular purpose). Expenditures are commonly classified by administrative unit (e.g., ministry, agency), but supplementary information by economic and/or functional categories are usually presented as well. The year-end report is the government's key accountability document. It is audited by the supreme audit institution and released within a defined period of the end of the fiscal year. The year-end report shows compliance with the level of revenue and expenditures authorised by Parliament in the budget. Any in-year adjustments to the original budget should be shown separately. The presentation format of the year-end report mirrors the presentation format of the budget (although sometimes in a more aggregated form).

'Unlabelled expenditure' refers to unplanned spending and was estimated through modelling techniques, based on a top-down budgetary procedure. Starting from overall aggregated expenditures, this procedure estimates the proportion causally attributable to drug use (Unlabelled Drug-related Expenditure = Overall Expenditure × Attributable Proportion). For example, to estimate the prison drug-related expenditures in a given country, two elements would be necessary: the overall prison expenditures in the country for a given fiscal year, and the attributable proportion of inmates due to drug-related issues. The product of the two will give a rough estimate that can be compared across different countries.

This twofold approach aimed to provide more comprehensive and accurate estimates of public spending in tackling drugs and drug addiction Europe-wide. While figures should be used with caution, estimates from reporting countries, extrapolated to European level, arrived at a total cost of drug-related public expenditure in 2005 of EUR 34 billion (labelled and unlabelled). This represents an average expenditure of EUR 60 per European citizen per year [2].

Of the total cost identified, only 7% (EUR 2.42 billion) was labelled expenditure [2]. This is somewhat paradoxical because from a drug policy perspective labelled expenditure is more relevant than unlabelled expenditure; labelled expenditure is proactive, in that it is linked to the achievement of specific policy aims, while unlabelled expenditure is reactive, in that it arises as a result of drug misuse, such as enforcement or health costs [3]. In any case, as discussed by Reuter [4], the "drug budget" (i.e. labelled expenditure) aimed at reducing drug use and related problems is a useful description of a nation's drug policy. Cross-country comparisons of their levels and composition can certainly be of use to policy decision makers.

The main challenge of comparing budget expenditure for international benchmarking across countries is consistency of reporting. In this respect, ten countries (Czech Republic, Ireland, France, Luxembourg, Hungary, Poland, Portugal, Slovakia, Finland, and the United Kingdom) yield labelled expenditure classified according to a common categorisation system: the international Classification of the Functions of Government (COFOG). COFOG is a detailed classification of the socioeconomic objectives that government units aim to achieve through a range of outlays (e.g. health, education, social protection, public order and safety, among others) [5]. Overall, of the total labelled expenditure categorised by these countries, 86% (EUR 1.88 billion) came within two government functions: Health (66%) (i.e. medical products, outpatient services, hospital services, public health services, R&D) and Public Order and Safety (POS) (20%) (i.e. police services, law courts, prisons) (Table S1, Additional file 1). The rest of the expenditures (not considered in the present report) went to General Public Services (5%), Housing and Community Ammenities (1.71%) Education (0.88%), Economic Affairs (0.62%), Social Protection (0.61%), Defence (0.05%), or were not appropriately classified (4.20%) [2].

By country, the average share of GDP was 0.023% for Health, and 0,013% for POS. However, these shares varied considerably across countries, ranging from 0.00033% in Slovakia, up to 0.053% of GDP in Ireland in the case of Health, and from 0.003% in Portugal, to 0.02% in the UK, in the case of POS; almost a 161-fold difference between the highest and the lowest countries for Health, and a 6-fold difference for POS (Table S1, Additional File 1). Why do Ireland and the UK spend so much in Health and POS, or Slovakia and Portugal so little, in GDP terms?

To respond to this question and to make a more comprehensive assessment of labelled drug-related public expenditure across countries, this study described and compared Health and POS spending per capita (response variable/s) and GDP per capita (explanatory variable) in the 10 reporting countries through linear regression analysis. All variables were transformed into natural logarithmic values to have them normally distributed and for interpretability purposes. Expenditures and GDP were expressed in 2005 Euro Purchasing Power Parity [6].

**Figure 1:**
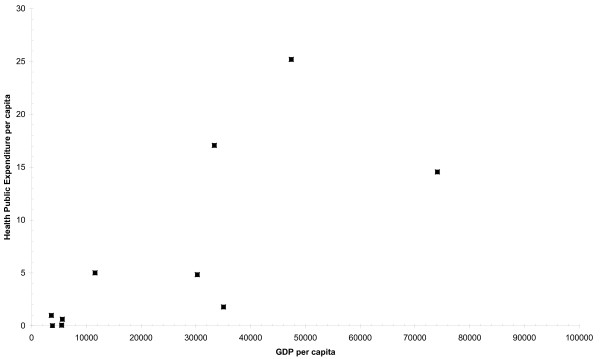
**Labelled Drug-Related Expenditure (Health) and GPD per capita, in Europe (PPP EUR 2005)**.

**Figure 2:**
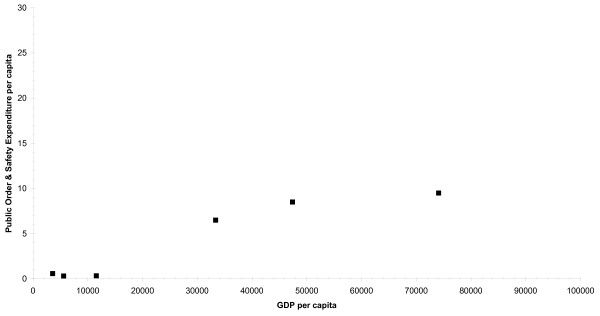
**Labelled Drug-Related Expenditure (Public Order and Safety) and GPD per capita, in Europe (PPP EUR 2005)**.

The results show the positive association between the GDP per capita and Health and POS spending per capita (Figures 1 and 2, respectively) across the 10 countries analyzed (Figures 1 and 2, respectively). There is an overall clear tendency for countries with higher GDP to spend a greater proportion of their GDP on Health (Pearson's r = 0.81, n = 10, exact 2-side p = 0.0032), and POS (Pearson's r = 0.91, n = 6, exact 2-side p = 0.0653), suggesting that GDP is an important determinant of labelled drug-related public expenditure in a country. The percentage change in Health and POS labelled expenditures per capita due to a one percent increase in GDP per capita (the income elasticity of demand (IED)) was estimated to be 1.78% (R^2 ^= 0.66, SEE = 1.56) and 1.23% (R^2 ^= 0.82, SEE = 0.79) respectively. The IED indicates how sensitive is the demand for a good to an income change [7]; if IED is greater than one, demand for the item is considered to have a high income elasticity. Luxury goods (e.g. jewels) are said to have high income elasticity: as people become wealthier, they will buy more and more of the luxury good. In economics, a luxury good is any type of good for which demand increases more than proportionally as income rises [8]. In our case, being highly "income" elastic, Health and POS expenditures can be considered luxury goods; as a country becomes wealthier it spends proportionately more on drug-related health and public order and safety interventions.

The results confirm the importance of GDP as a determinant of Health and POS spending, and suggest that this effect is higher on POS than in Health; GDP accounted for 82% of the variation in POS expenditure, and 66% of the variation in Health.

The variations in overall health care expenditure and GDP have already been extensively explored in the literature [9]. Earlier studies were based, like the present study, on cross-sectional approaches for particular years [10,11]. Other studies have relied on the analysis of dynamic data, or time series panel data to determine the relation between GDP and health care expenditure [12-14]. Overall, results have confirmed the importance of GDP as a determinant of health spending, with an estimated income elasticity of demand above unity.

The present study suggests the confirmation of an intuitive expectation: the elements of economic wealth of a nation and public investment in the problems of illicit drugs are interdependent. This fact has important implications for policy making by forcing decision makers to take budgetary effects into account when defining drug strategies and action plans, something that currently is not appropriately implemented in the EU.

Nevertheless, caution is needed when considering the conclusions obtained from the results of the regression analysis in this study; there are a series of limitations that might preclude the conclusions reached.

First, labelled drug-related public expenditure is a component of GDP, thus, all things being equal, an increase of public drug-related expenditure will raise GDP by the same amount. Under these circumstances, a positive correlation can be expected between GDP and public expenditure on drugs, due to the fact that one variable includes the other (part-whole correlation), even when there would not be association between them.

Second, the calculations made in the study are based on the assumption that drug-related public expenditure depends on a country's GDP. Is this hypothesized causal relationship plausible? The regression results found do not necessarily establish a causal relationship between GDP and public expenditure. Although the statistical computations used to produce the estimated measure of association are appropriate for descriptive purposes, the estimate itself may be biased. Bias may result from not considering in the analysis other variables that could account for the observed association, such as the size of the drug problem in the country, the political orientation of government, or the model of state. The resulting biases can distort the true value of the correlation coefficient and lead to a false conclusion on the relationship between the two variables involved in the analysis. Should this be the case, a deeper assessment of the plausibility of the causal relationship between GDP and labelled expenditure must be performed.

Third, only 10 countries were included in the analysis. Unfortunately, studies on public expenditure require a significant amount of analytical work for the labelled component, and require a certain degree of creativity as far as non-labelled expenditure is concerned. Altogether, this meant that comprehensive approaches to precisely estimate public expenditure were beyond the technical, resource or human capabilities of some national focal points in the EU. The reporting countries cannot be considered a representative sample of the 27 EU member states; thus, the results obtained are only applicable to the countries analyzed, and no generalizations can be made to the remaining countries. This situation should be resolved over time by providing simple, clear and straightforward guidelines on how to proceed in identifying labelled and unlabelled expenditure across countries in the EU. The EMCDDA can play a leading role in this action by compiling the different strategies available for identifying expenditure, based on the experiences reported by the countries who have already carried out this exercise.

Expansions of the analyses presented here can obviously be made by adding countries, by reflecting other type of expenditures (e.g. private expenditures), by including other explanatory variables in the model, and by considering additional years of reference (e.g., panel data). The result will be a greater insight into the understanding of differences in labelled drug-related public expenditures in different countries. In addition, this could help explain how they influence the quality and quantity of drug-related interventions, and which of them perform better in terms of cost-effectiveness.

## Competing interests

The author declares that they have no competing interests.

## Author's information

The author was a staff member of the European Monitoring Centre for Drugs and Drug Addiction (EMCDDA) (Scientific Analyst - Economic Analysis). He currently is a Distance Learning Tutor and Examiner -Public Health, and Health Systems Management PGDip/MSc- at the London School of Hygiene and Tropical Medicine (London, since 2007), and Associate Editor of Health and Quality of Life Outcomes journal (http://www.hqlo.com/, since 2009).

He received a Master of Business Administration (ESADE, Madrid, 2005), a PGDip in Health Economics (University of York, 2004), a Master in Public Health (Autonomous University of Barcelona UAB, 1999), and a PhD in Public Health and Biomedical Research Methodology (UAB, 1995).

## Supplementary Material

Additional file 1 Table S1: Labelled Drug-Related Public Expenditure and GPD per capita, in Europe in 2005. Click here for file
