# Supplementary material for: Labelled drug-related public expenditure in relation to gross domestic product (gdp) in Europe: A luxury good?
Source: Subst Abuse Treat Prev Policy. 2010 May 17;5:9. doi: 10.1186/1747-597X-5-9 (PMC2881082; doi:10.1186/1747-597X-5-9)
Supplement: Additional file 1 — Table S1: Labelled Drug-Related Public Expenditure and GPD per capita, in Europe in 2005. [file 1747-597X-5-9-S1.DOC]

**Table S1. Labelled Drug-Related Public Expenditure and GPD per capita, in Europe in 2005.**

| **Country** | **Labelled Public Expenditure** | | **Labelled Public Expenditure as a percentage of GDP** | | **Labelled Public Expenditure**  **per capita** | | **GDP per capita**  **(2005 PPP EUR)** |
| --- | --- | --- | --- | --- | --- | --- | --- |
|  | **Health**  **(EUR million)** | **Public Order and Safety**  **(EUR million)** | **Health**  **(%)** | **Public Order and Safety**  **(%)** | **Health**  **(2005 PPP EUR)** | **Public Order and Safety**  **(2005 PPP EUR)** |  |
| **Czech Republic** | 11.1 | 5.8 | 0.011 | 0.006 | 0.62 | 0.32 | 5,595 |
| **Ireland** | 85.8 | 29.0 | 0.053 | 0.018 | 25.19 | 8.51 | 47,405 |
| **France** | 275.1 | n.a | 0.016 | n.a | 4.84 | n.a | 30,235 |
| **Luxembourg** | 5.9 | 3.9 | 0.020 | 0.013 | 14.55 | 9.50 | 74,080 |
| **Hungary** | 0.9 | n.a | 0.001 | n.a | 0.05 | n.a | 5,459 |
| **Poland** | 67.0 | 40.0 | 0.027 | 0.016 | 0.98 | 0.59 | 3,586 |
| **Portugal** | 64.6 | 4.4 | 0.043 | 0.003 | 5.01 | 0.34 | 11,563 |
| **Slovakia** | 0.1 | n.a | 0.0003 | n.a | 0.01 | n.a | 3,787 |
| **Finland** | 8.0 | n.a | 0.005 | n.a | 1.78 | n.a | 35,017 |
| **United Kingdom** | 923.3 | 352.0 | 0.051 | 0.020 | 17.06 | 6.51 | 33,352 |

**n.a. – not available**

**PPP – Purchasing Power Parity**

**Source: EMCDDA (2008), and Eurostat.**
